# Reproductive Behaviour of 150-Gy-Treated Female *Lobesia botrana* (Lepidoptera: Tortricidae)

**DOI:** 10.3390/insects13070600

**Published:** 2022-06-30

**Authors:** George Saour, Ali Hashem, Iyad Jassem

**Affiliations:** Atomic Energy Commission of Syria, Damascus P.O. Box 6091, Syria; ahashem@aecs.sy (A.H.); ijassem@aecs.sy (I.J.)

**Keywords:** European grapevine moth, gamma irradiation, insect control method, sperm precedence

## Abstract

**Simple Summary:**

Environment-friendly and sustainable insect pest control approaches such as the sterile insect technique/inherited sterility (SIT/IS) have been proposed against the European grapevine moth *Lobesia botrana*. Reproductive behaviour, such as oviposition, mating ability, and multiple mating of 150 Gy-treated females, was studied. Moreover, the last-male sperm precedence (P2 value) and male’s flight response to treated females were also investigated. The present study aims to provide basic information which is essential to ensure successful implementation of SIT/IS as part of an integrated approach for the management of *L*. *botrana*.

**Abstract:**

The sterile insect technique/inherited sterility (SIT/IS) has been suggested as an eco-friendly control tactic for area-wide integrated pest management approaches in order to control the European grapevine moth, *Lobesia botrana*. This study assessed the effects of an irradiation dose of 150 Gy administered to newly emerged female moths on their egg laying behaviour and mating ability at different ages at mating. Moreover, the effects of multiple mating on the mating ability of treated females, pattern of sperm precedence in twice-mated females and the flight response of treated and untreated males to treated and untreated calling females were also investigated. Females treated with 150 Gy initiated calling in a way similar to untreated females. When treated females were paired with untreated males, the mean number of eggs oviposited per female during 6 days was reduced (59.6 and 82.8 eggs/female, respectively), as was their mating ability and multiple mating compared with untreated females. The proportion of offspring fertilized by the second of the two males to mate with the female or last-male sperm precedence (P2 value) constituted 97% of the eggs, suggesting that the second male mate fathered the most offspring. The outcome of this work could be viewed as an integrated approach for improving effectiveness and enabling successful implementation of a SIT/IS program against *L*. *botrana.*

## 1. Introduction

The European grapevine moth *Lobesia botrana* (Denis and Schiffermuller) (Lepidoptera: Tortricidae) is one of the most destructive pests of grapes and berries in Europe, the Middle East, and Africa [1], as well as in the economically important grape-growing regions of Latin America, where it was accidentally introduced [2]. The sterile insect technique/inherited sterility (SIT/IS) has been proposed as a potential control tactic for use in area-wide integrated pest management (AW-IPM) approaches against this pest. It should be noted that the release of *L*. *botrana* males with IS suppress wild populations to a greater extent than an equal number of fully sterile males [3]. AW-IPM programs that include an SIT component can efficiently manage lepidopteran pest species [4]. Many authors have addressed the characteristic cytogenetic features of Lepidoptera, as well as their high resistance to ionizing radiation. The latter property resulted in the development of the inherited sterility IS method, a SIT-derived technology, which is currently used to successfully suppress Lepidoptera populations [5].

SIT/IS requires mass-rearing of the species, the use of ionizing radiation to sterilize the adults produced, and release of the treated adults into the target area. Mating of an irradiated male moth with a wild virgin female moth will induce egg sterility in the native population that will result in a gradual decline of the density of the wild population with each generation [4].

There are no efficient, robust, and appropriate approaches to mass separate the sexes of most lepidopteran species, so the release of only males is not possible for most of them, and for *L*. *botrana* in particular. Any SIT/IS program against this pest will thus require the release of both sexes [6,7]. In such an approach, the irradiation dose that is applied should confer full sterility in the females, and, as males are usually more radiation resistant, partial sterility in the male. However, the sterility is inherited by the siblings of the partially sterile male and unsterilized wild female mating, resulting in the F1 males being completely sterile [8,9]. It needs to be noted that the use of the SIT/IS against *L*. *botrana* has so far only been used in Chile on a small scale [10]. Hitherto, however, no studies have investigated the effects of a 150 Gy treatment administered to *L*. *botrana* female moths on several aspects of their mating behaviour and on the field release of irradiated male and female *L*. *botrana* in a vineyard. Stringer et al. [11] reported that there may be a benefit from releasing both sexes over male-only for *Epiphyas postvittana* (Lepidoptera: Tortricidae) with females acting as a sperm sink, and this benefit may be somewhat mitigated by multiple mating in male moths. Moreover, Ikegawa et al. [12] suggested that bisexual release of sterile males and females can be a compatible measure to efficiently suppress wild pest populations.

Females can mate more than once during their lifespan and polyandry (about 30% of the population) is a recessive, autosomally inherited trait in wild *L*. *botrana* populations. *L*. *botrana* females mate only once during 24 h and have been observed to start calling immediately after the beginning of dusk and until the end of the scotophase [13,14]. Accordingly, the pattern of sperm precedence may influence the outcome of the SIT/IS program applied against this species. Sterile males are known to be of lesser quality than wild males due to colonization and irradiation constraints. Thus, the dynamics of sperm could negatively affect the effectiveness of the SIT/IS because female receptivity is associated with the quality of the first male ejaculate [15].

In a previous study, Saour [3] studied the effects of various doses of gamma radiation on the fecundity and fertility of *L*. *botrana*. Moreover, the study proposed a dose of 150 Gy at which females became fully sterile when mated with fertile males. However, there are no data available of the effect of treating females with a dose of 150 Gy on their oviposition behaviour and the impact of their mating with untreated and 150 Gy-treated males. Moreover, male sperm precedence (P2 value) in twice-mated *L*. *botrana* females has not yet been studied. Therefore, this study aims to determine (1) the response of 150 Gy-treated and untreated males to 150 Gy-treated calling female moths assessed as their flight ability in flight assessment cages, (2) female mating ability at different age at mating, (3) the effect of multiple mating on the mating ability, (4) the pattern of sperm precedence in twice-mated females, and (5) the oviposition behaviour of treated and untreated females.

## 2. Materials and Methods

All insects were reared on a semi artificial diet as outlined in Thiéry and Moreau [16] and Saour [3]. Laboratory-rearing procedures and all the experiments were carried out at a constant temperature of 25 ± 1 °C, 60 ± 10% RH and a photoperiod of 15: 8 h L: D + 1 h of dusk (calling, mating and egg-laying, is principally observed at dusk).

### 2.1. Moth Irradiation

The laboratory reared *L. botrana* adults were exposed to an irradiation dose of 150 Gy in a ^60^Co irradiator (Issledovatel Gamma Irradiator, Techsnabexport Co., Ltd., Moscow Russia) at a dose rate of 9.8 Gy/min. The moths (<18 h old) were sexed and placed individually in small clear plastic tubes, 1 cm in diameter and 8 cm high, prior to irradiation treatment. The dose uniformity ratio (max:min of the received dose) was about 1.14 and the absorbed dose was calibrated using Fricke dosimetry.

### 2.2. Response of Treated and Untreated Male Moths towards Calling 150 Gy-Treated and Untreated Female Moths

The experimental flight cage described by Saour [7] was used to assess the flight performance of the untreated and treated *L*. *botrana* male adults responding to 150 Gy-treated and untreated calling females. Cohorts of newly emerged (<24 h old) male and female *L*. *botrana* moths were irradiated with a dose of 150 Gy. Treated and untreated male moths (*n* = 25 for each) were held at 4 °C for 3 min for immobilization and then placed on the bottom of the flight cage (male compartment). One-day-old 150 Gy-treated and untreated virgin females (*n* = 5 for each) were confined inside a small cylindrical plastic mesh box that contained a cotton wick soaked in 5% sucrose solution and placed in the cage (female compartment). The number of males that had flown through the opening at the 45 cm height into the female compartment was recorded after 24, 48, 72, and 96 h. Two colours of fluorescent powders (provided by the Insect Pest Control Laboratory, FAO/IAEA, Seibersdorf, Austria) were used to mark and distinguish between the 150 Gy-treated and untreated male moths.

The bioassay in the experimental flight cage to study the male flight ability and response to calling female moths included two combinations, as shown in Table 1. The experiment was replicated six times for each combination tested.

### 2.3. Female Mating Ability at Different Age at Mating after Irradiation

One-day-old adult females (*n* = 300) were irradiated with a dose of 150 Gy and were kept in transparent Petri dishes for different lengths of time after the treatment before offering a mating opportunity with newly emerged untreated males. All of the females were provided with a 5% sucrose solution as a food source. The treated female moths were divided into seven groups, each consisting of 30 females. The females of group 1 were mated on the day of the irradiation treatment, the females of group 2 were mated 1 day after irradiation, etc., and finally the females of group 7 were mated 6 days after irradiation. One hour before dusk on the day of mating, each virgin female moth was placed with two 2 day old virgin male moths in 9 cm Petri dishes for 24 h. Seven groups of untreated females (30 in each one) aged 1, 2, 3, 4, 5, 6, and 7 days old were considered as the controls. The control females were confined with virgin male moths in the same manner as the treated females. In each treated and untreated group, the oviposited eggs were removed daily, counted, and the percentage egg hatch assessed (for the untreated control group only). At the end of the experiment, all of the females were dissected and examined for the presence of a spermatophore in the *bursa copulatrix*.

### 2.4. Effect of Multiple Mating on the Mating Ability of 150 Gy-treated Female Moths

One-day-old adult females (*n* = 45) were irradiated with a dose of 150 Gy, and then paired singly with newly emerged untreated male moths (0–18 h). The males were removed after 24 h and the females were kept for oviposition. One-day-old virgin untreated male moths were confined with the same females the next day for 24 h and the same procedure was followed for 7 successive days. The females of the control group (*n* = 45) were subjected to the same mating protocol. Therefore, the experiment consisted of seven different groups of treated and untreated female moths each offered a fresh male every day for 7 consecutive days. All of the eggs oviposited by the individual treated and untreated females were counted and the egg hatch was determined. Dead female moths were preserved in 70% ethanol and later dissected under a stereo microscope for the presence of spermatophores in their *bursa copulatrix*.

### 2.5. Sperm Precedence in Twice-Mated Females

Newly emerged males (<18 h old) were treated with 150 Gy and then divided into four groups (50 males/group). The males of each group were individually paired with newly emerged female moths; after 24 h, the first males were replaced with new males for another 24 h, and then the second males were removed. The following experimental groups with twice-mated females were obtained (first 150 Gy-treated ♂, second untreated ♂), (first untreated ♂, second 150 Gy-treated ♂), (first 150 Gy-treated ♂, second 150 Gy-treated ♂), and (first untreated ♂, second untreated ♂). The females were kept for oviposition until death. All of the eggs were collected, counted, and allowed to hatch in order to determine the fecundity and fertility. After death, the female moths (in each group) were dissected and examined for the presence of a spermatophore in the *bursa copulatrix*. Only females with two spermatophores (twice-mated females) were taken into consideration. The percentage of egg fertilized by sperm from the second mate (P2 value, second male paternity) was calculated according to the Ueno and Ito [17] formula, as follows:

*x* = 100 (*a* − *c*)/(*b* − *c*) (%)

where, *x* is P2 value, *a* is the observed percentage of egg hatch in treated—untreated matings, and *b* and *c* are the percentage of egg hatch in untreated—untreated matings and in treated—treated matings, respectively.

### 2.6. Temporal Oviposition Dynamics of 150 Gy-Treated and Untreated Female Moths

Newly emerged female moths (0–18 h old) were treated with 150 Gy gamma radiation (*n* = 75) and the same number of females of the same age were treated and considered as the control group. Treated and untreated female moths were paired individually with 1 to 2 day old untreated virgin male moths in transparent plastic Petri dishes (9 cm diameter, 1 cm high). The moths were provided with water *ad libitum* through a soaked cotton wick placed in the petri dish with them. The inner Petri dish surface served as the oviposition substrate. For both the treated and untreated group, the females were left with the male moths for 24 h. The females were kept for oviposition until death. The deposited eggs were checked daily (eggs that were oviposited during the previous night were identified by drawing circles on the outside of the lid of the Petri dish with a marker) and counted (no hatch was expected for eggs oviposited by treated females). Only female moths that oviposited a sufficient number of eggs (>15 eggs) during their lifetime were considered, so as to stabilize the variance. The experiment was replicated three times with 25 females/replicate.

### 2.7. Statistical Analysis

Data analyses were carried out using Stat-view program [18] at the 5% level (*p* ≤ 0.05). Numerical data are expressed as mean ± standard deviation (SD). One-way analysis of variance (ANOVA) followed by Fisher’s protected least significant difference test (PLSD) were conducted for all possible mean comparisons. A normal approximation test (Z) was used to make comparisons between the percentages.

## 3. Results

### 3.1. Response of Treated and Untreated Male Moths towards Calling 150 Gy-Treated and Untreated Female Moths

Treated females were able to initiate calling, as did the untreated females (based on male moth’s response), although they were irradiated with a sterilizing dose of 150 Gy (Table 2). The response of the untreated and treated males flying towards the calling females was influenced by whether the female was treated or not, and the age of the calling females. The greatest proportion of treated and untreated males flying into the female compartment was observed on the second day of the experiment; the greatest percentage of male non-flyers was recorded for 150 Gy-treated male moths that were responding to 150 Gy-treated females (27%; F3,20 = 37.6; *p* ≤ 0.05). Furthermore, the untreated males were the most responsive to the calling untreated (10.5 vs. 15.1%, respectively) and treated females (14.5 vs. 27.0%, respectively), as shown by the fewer non-flyers in the cage (Table 2).

### 3.2. Female Mating Ability at Different Age at Mating after Irradiation

**Figure 1:**
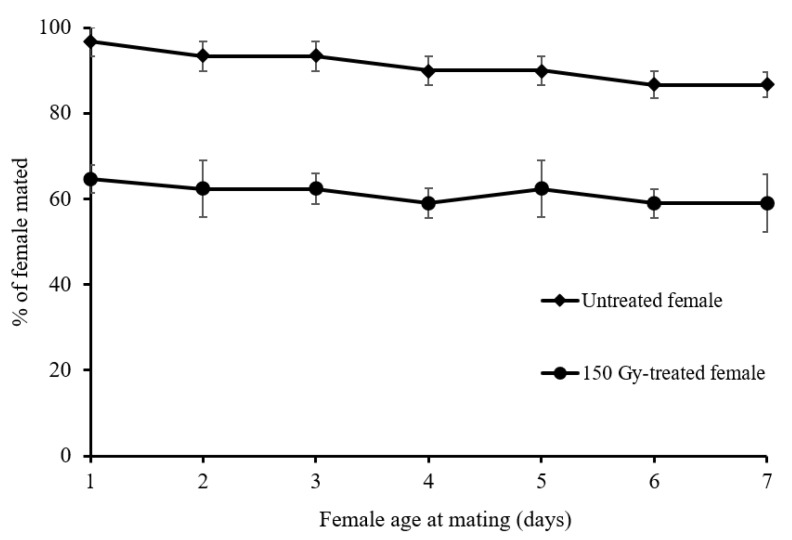
Percentage of copulations (±SD) of untreated and 150 Gy-treated *Lobesia botrana* females of different ages with untreated virgin males.

The results in Figure 1 reveal that female age at mating (up to 7 day old females) had no effect on the mating ability of treated or untreated females, although a noticeable difference in the mating ability between treated female and untreated moths was observed regardless of the female’s age (e.g., for 1-day-old females F1,4 = 5712.6; *p* ≤ 0.05).

### 3.3. Effect of Multiple Mating on the Mating Ability of 150 Gy-Treated Female Moths

Table 3 shows that the percentage of mating in untreated females were 95.6% and decreased to 60.0% in treated female moths, despite exposing them to new virgin males every 24 h for 7 consecutive days (Z = 3.97; *p* ≤ 0.05). There were no female moths, for both the treated and untreated groups, that mated more than three times. The percentage mating ability during the first mating opportunity (60.4 and 66.7%) was significantly higher than the mating ability during the second (23.3 and 25.9%) and third (7.4 and 16.3%) mating opportunities for the treated and untreated females, respectively. Significantly, more untreated females mated with a virgin male moth during the first mating opportunity compared with the treated moths, but there were no significant differences in mating ability between the two groups for the second and third mating opportunity. Fecundity of the treated moths was significantly lower than that of the untreated females, but was similar for both groups for the three matings. Egg hatch was in the untreated control group similar for the three matings (≈80%), while no egg hatch occurred for the treated group (Table 3).

### 3.4. Sperm Precedence in Twice-Mated Females

Eggs oviposited by untreated female moths that had mated twice with untreated males and twice with 150 Gy-treated males showed an average hatch of 79.7% and 40.7%, respectively (Table 4). Female moths that had mated first with an untreated male and then with a150 Gy-treated male oviposited eggs, of which 42.2% hatched. Conversely, females that mated first with a 150 Gy-treated male and then with an untreated male produced eggs, of which 78.7% hatched (F3,20 = 371; *p* ≤ 0.05) (Table 4). According to the Ueno and Ito [17] method, a complete last-male sperm precedence will be obtained if the P2 value is equal to 1 (P2 = the proportion of offspring sired by the second or last male to mate). Thus, by applying the aforementioned method, a P2 value of 0.97 was obtained. This P2 value suggests that the second male mate fathered 97% of the offspring, while 3% of the offspring were sired by the first male mate.

### 3.5. Temporal Oviposition Dynamics of 150 Gy-Treated and Untreated Female Moths

**Figure 2:**
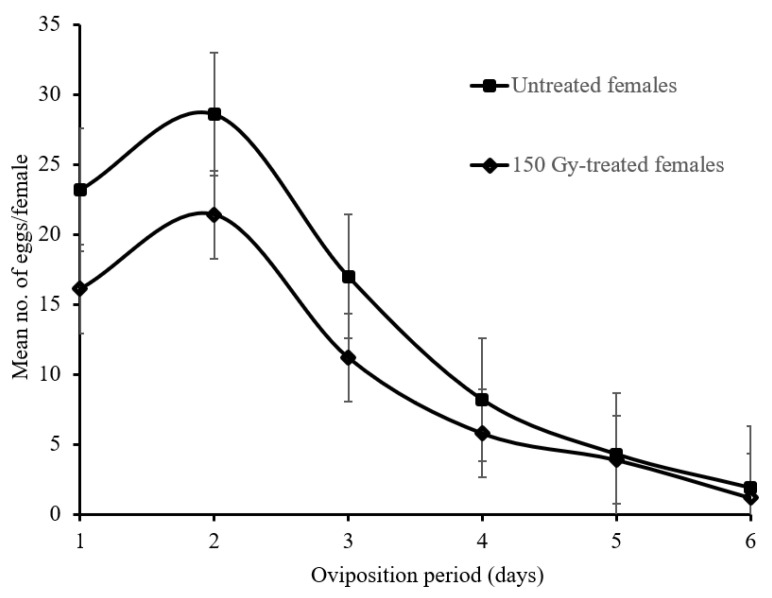
Mean number (±SD) of eggs laid by untreated and 150 Gy-treated *Lobesia botrana* females mated with untreated males according to the day of oviposition.

Figure 2 shows that the mean number of eggs oviposited significantly decreased with time or with female age for both the treated and untreated female moths (F5,144 = 129.7; *p* ≤ 0.05 and F5,144 = 161.5; *p* ≤ 0.05 for treated and untreated females, respectively). The mean number of eggs oviposited by treated female moths during the entire oviposition period was significantly lower (F2,52 = 101.5; *p* ≤ 0.05) compared with the untreated females (i.e., 59.6 ± 10.5 and 82.8 ± 11.7 eggs/female, respectively). Both the treated females and the untreated female’s, oviposited the maximum of eggs during the first 2 days of the oviposition period, while relatively few eggs were oviposited in the following 3 days (Figure 2).

## 4. Discussion

The field competitiveness of gamma irradiated male and female moths and their mating ability are important factors that influence the success of SIT/IS [19,20]. The laboratory flight bioassay assessed the response of 150 Gy-treated and untreated males to the calling of treated and untreated female moths. In our experimental flight cage, it is presumed that the sex pheromone emitted by *L*. *botrana* females would be the main trigger for the male flight response [6]. A dose of 150 Gy did not negatively affect virgin female pheromone emission, as there were no significant differences in terms of the flight ability of untreated males towards treated or untreated calling females. It is noteworthy to mention that a similar finding was reported by Suckling et al. [21] who examined the effect of gamma irradiation (100–500 Gy) on *Teia anartoides* (Walker) (Lepidoptera: Lymantriidae) female moths used to bait traps to attract male moths in the field. However, most of the non-flyer male moths were found in the 150 Gy-treated group when challenged with treated females. Saour [7] reported similar results regarding the perception of *L*. *botrana* female pheromone by 150 Gy-treated males.

Our findings that both virgin 150 Gy-treated and untreated female showed relatively similar calling behaviour suggest that if both treated sexes are released together, the treated and feral males may court the treated females, and the released females could attract both feral and 150 Gy-treated males.

Many factors influence the mating process in Lepidoptera, including the time of calling, the release of pheromones, and the subsequent sensing of these chemical cues by males [22]. Our results indicate that the mating ability of females aged between 1 and 7 days was similar, irrespective of whether the females were treated with 150 Gy or were untreated. This result is in accordance with earlier findings of Torres-Vila et al. [23] who showed that female age (up to 12 days old) did not affect the mating ability of *L. botrana*. Virgin female *L*. *botrana* maintained calling activity and receptivity, regardless of their age, and the pheromone gland did not degenerate with the ageing of the females [24,25]. On the contrary, the mating ability of *Cydia*. *Pomonella* L. (Lepidoptera: Tortricidae)*, Plutella xylostella* (L.) (Lepidoptera: Plutellidae), *Phthorimaea operculella* Zeller (Lepidoptera: Gelechiidae), and *Phauda flammans* (Walker) (Lepidoptera: Phaudidae) was significantly reduced with female age [20,26,27,28].

The noticeable difference in mating ability between 150 Gy treated and untreated females, regardless of the age of the female at mating day, suggests that the lower mating ability of treated females is related to the radiation treatment. Similar effects have been reported for *P*. *operculella* and *C*. *pomonella* [19,20].

When *L*. *botrana* treated females were allowed to mate with newly emerged males for 7 consecutive days, about two-thirds of them mated once, one-quarter mated twice, and the remaining (7.4%) mated three times. Regardless of differences in mating ability, almost similar results were obtained for the untreated females (Table 3). The numbers of eggs oviposited by either 150 Gy-treated or untreated females were comparable, irrespective whether the female mated once, twice, or three times. This would indicate that once-mated females received a sufficient quantity and quality of sperm and male accessory gland secretions to fertilize their full egg complement during the first mating. Many studies argue that female moths that did not receive adequate effective sperm during the first mating would seek additional matings [26,29].

Untreated and 150 Gy-treated *L*. *botrana* females, similar to *P*. *operculella* females, did not mate more than three times when they were paired with virgin males for seven successive days. In contrast, *C*. *pomonella* females mated up to five times when they were exposed to newly emerged males for 7 consecutive days [19,20].

Multiple mating is a widespread phenomenon in Lepidoptera and polyandry has evolved, allowing different males to fertilize the eggs of a female. This allows females to simultaneously gain additional nutrients and/or genetic benefits [30,31,32,33]. In *L*. *botrana*, the current study showed that the last male to mate with the female sired most of her offspring, as the P2 value was close to 1 (0.97). In other words, sperm from successive matings in *L*. *botrana* did not compete in a random manner, but the sperm transferred during the most recent mating was predominately utilized to fertilize the eggs. Complete last-male sperm precedence was found in many economic lepidopteran species, including *Ephestia kuehniella* (Zeller) (Lepidoptera: Pyralidae), *Spodoptera litura* (F.) (Lepidoptera: Noctuidae), and *P*. *operculella* [34,35,36].

The pattern of sperm precedence in *L*. *botrana* may positively or negatively affect the outcome of a SIT/IS against *L*. *botrana*, as the effect of a 150 Gy-treated male mated with a wild virgin female would be negated by a second mating with a wild male and vice versa. Therefore, the most effective way to mitigate the possibility of a second mating with wild males is through the use of a larger 150 Gy-treated moths to wild males overflooding ratio [37]. Another concern is whether wild females mated with 150 Gy-treated males are more likely to re-mate with untreated males, than wild females mated first with wild males. These potential negative affects the SIT/IS against *L*. *botrana* need to be studied and would require that females can actively discriminate between untreated and 150 Gy-treated males.

Our results indicate that the fecundity of treated *L*. *botrana* females were reduced compared with untreated females. In general, oviposition behaviour varies among insect species; it is controlled by a genetic pathway formed by oviposition-related genes, and influenced by physiological pressure from the oogenesis process and oocyte formation [38,39]. The untreated and treated females used in this study showed an average fecundity of 83.2 ± 16.5 and 59.6 ± 13.6, respectively. In both cases, gravid females oviposited approximately 63% of their eggs in the 2 days of the oviposition period. Wainwright et al. [40] reported that *P. xylostella* females oviposited 90% of their eggs during the first 3 days after emergence. In our study, the mean number of eggs increased on day 2 and decreased thereafter until the female had died (Figure 2). A comparable result has been observed in *C*. *pomonella* and *P*. *operculella* female moths [19,20,41]. In light of our findings, the availability of a significant number of infertile eggs deposited by treated females (≈60 eggs/female) could enhance the impact of SIT/IS against *L*. *botrana* wild populations. These non-fertile eggs can be considered as a benefit in a program where natural enemies are combined with SIT/IS, because they can provide additional hosts for egg parasitoids and be a food source for predators [42].

For the efficiency of the SIT/IS program against the Lepidoptera pest in general and *L*. *botrana* in particular, the positive or negative effects of a male-only release, compared with that of a bisexual release, have not been yet determined. Thus, the data presented herein provide useful information that is essential to ensure successful implementation of the SIT/IS as a part of an integrated approach for the management of *L*. *botrana*.

## 5. Conclusions

In any SIT/IS program to manage *L*. *botrana*, 150 Gy-treated male and female moths would have to be released as there is no robust and practicable genetic sexing method in Lepidoptera. Therefore, this study highlights the effects of the irradiation dose of 150 Gy on *L*. *botrana* female’s reproductive behaviour. The P2 value in this species is about 0.97; thus, an overflooding ratio of 150 Gy-treated male and female moths to wild insects should be considered to induce inherited sterility into the wild population and to mitigate the effect of female re-mating with wild males. Further studies about the mating competitiveness of 150 Gy-treated *L*. *botrana* male and female moths or 150 Gy-treated male moths only, with untreated moths are still essential to the SIT/IS effectiveness against this lepidopteran pest. However, the data generated in this study have great practical value and will help in implementing SIT/IS against *L*. *botrana* populations.

## Figures and Tables

**Table 1:** Combinations of *Lobesia botrana* male and female moths in the experimental flight cage.

Male Compartment	Female Compartment
Combination 1 Untreated ♂ + 150 Gy-treated ♂ (*n* = 25 for each)	Untreated ♀ (*n* = 5)
Combination 2 Untreated ♂ + 150 Gy-treated ♂ (*n* = 25 for each)	150 Gy-treated ♀ (*n* = 5)

**Table 2:** Mean percentages (±SD) of untreated and 150 Gy-treated *Lobesia botrana* males succeeding in flying to virgin untreated and 150 Gy-treated calling females in the calling female compartment in a flight assessment cage during 4 successive days. The mean percentages (±SD) of males that failed to fly into the female compartment is consigned in the column farthest to the right.

Type of Female/Male Tested *	% of Males That Flew into Females Compartment on the Indicated Day				% of Non-Flying Males
	1st Day	2nd Day	3rd Day	4th Day	
Untreated female/					
150 Gy-treated male	18.1 ± 3.8 B,b	43.3 ± 4.6 A,a	14.6 ± 4.8 B,a	8.9 ± 3.6 C,a	15.1 ± 6.7 B,b
Untreated male	23.4 ± 3.2 B,a	47.4 ± 2.7 A,a	12.7 ± 4.7 C,a	6.0 ± 3.3 D,a	10.5 ± 4.2 C,c
Treated female/					
150 Gy-treated male	13.2 ± 3.0 C,c	38.2 ± 2.0 A,b	16.0 ± 2.5 C,a	5.6 ± 3.2 D,a	27.0 ± 4.5 B,a
Untreated male	20.0 ± 2.5 B,a	44.6 ± 4.8 A,a	14.2 ± 3.8 C,a	6.7 ± 2.1 D,a	14.5 ± 3.0 C,b

* 25 male moths were used for each type of male/female tested. Means of 6 replicates. Means in each row followed by the same uppercase letter are not significantly different (*p* ≤ 0.05, Fisher PLSD); means in each column for each day followed by the same lowercase letter are not significantly different (*p* ≤ 0.05, Fisher PLSD).

**Table 3:** Effect of repeated mating on percentage of mating ability, fecundity and fertility of 150 Gy-treated and untreated *Lobesia botrana* females mated with untreated males.

Dose (Gy)	No. of Females	Mating Ability (%)	No. of Mating	No. of Mated Females (%)	Mean No. of Eggs/Female (±SD)	Mean % Fertility (±SD)
150	45	27/45 (60.0) b	1	18 (66.7) b	54.1 ± 9.0 b	0
			2	7(25.9) cd	56.8 ± 6.4 b	0
			3	2(7.4) e	55.5 ± 6.4 b	0
0	45	43/45 (95.6) a	1	26 (60.4) a	84.4 ± 6.4 a	82.2 ± 2.6 a
			2	10 (23.3) cd	83.4 ± 5.8 a	80.1 ± 1.9 a
			3	7 (16.3) cde	81.3 ± 4.4 a	79.9 ± 3.1 a

Percentages in columns followed by the same letters are not significantly different (*p* < 0.05, normal approximation test). Means in columns followed by the same letters are not significantly different (*p* < 0.05, Fisher PLSD).

**Table 4:** Mean percentage of egg hatch (±SD) and percentage of twice-mated females in *Lobesia botrana* females mated sequentially with 150 Gy-treated and untreated males.

Mating Sequence	Mean % Egg Hatch	Twice-Mated Females (%)	P2 Value
			0.97
Untreated–Untreated	79.7 ± 0.60 a	25.0 ab	
Untreated–Treated	41.2 ± 1.7 b	30.0 a	
Treated–Untreated	78.7 ± 2.3 a	20.0 b	
Treated–Treated	40.7 ± 2.4 b	25.0 ab	

Means in columns followed by the same letter are not significantly different (*p* < 0.05, Fisher PLSD). Percentage in columns followed by the same letter are not significantly different (*p* < 0.05, normal approximation test).

## Data Availability

Data are available upon request from the authors.

## References

[1] Thiéry D., Monceau K., Moreau J. (2014). Different emergence phenology of European grapevine moth (*Lobesia botrana*, Lepidoptera: Tortricidae) on six varieties of grapes. Bull. Entomol. Res..

[2] Scaramozzino P.L., Giovanni F.D., Loni A., Gisondi S., Lucchi A., Cerretti P. (2020). Tachinid (Diptera, Tachinidae) parasitoids of *Lobesia botrana* (Denis & Schiffermüller, 1775) (Lepidoptera, Tortricidae) and other moths. ZooKeys.

[3] Saour G. (2014). Sterile insect technique and F_1_ sterility in the European grapevine moth, *Lobesia botrana*. J. Insect Sci..

[4] Bourtzis K., Vreysen M.J.B. (2021). Sterile Insect Technique (SIT) and its applications. Insects.

[5] Marec F., Vreysen M.J.B. (2019). Advances and challenges of using the sterile insect technique or the management of pest Lepidoptera. Insects.

[6] Haff R., Ovchinnikova I., Liang P., Mahoney N., Gee W., Gomez J., Toyofuku N., Jackson E., Hnasko R., Light D. (2020). X-ray-based irradiation of larvae and pupae of the navel orangeworm (Lepidoptera: Pyralidae). J. Econ. Entomol..

[7] Saour G. (2016). Flight ability and dispersal of European grapevine moth gamma-irradiated males (Lepidoptera: Tortricidae). Fla. Entomol..

[8] Makee H., Saour G. (2004). Efficiency of inherited sterility technique against *Phthorimaea operculella* Zeller (Lepidoptera: Gelechiidae) as affected by irradiation of females. J. Veg. Crop. Prod..

[9] Potgieter L., van Vuuren J.H., Conlong D.F. (2012). Modelling the effects of the sterile insect technique applied to *Eldana saccharina* Walker in sugarcane. ORiON.

[10] FAO (2020). The Sterile Insect Technique for Use against the Devastating European Grapevine Moth in Chile. http://www-naweb.iaea.org/nafa/news/2018-developing-area-wide-SIT-chile.html.

[11] Stringer L.D., Sullivan N.J., Sullivan T.E.S., Mitchell V.J., Manning L.-A.M., Mas F., Hood-Nowotny R.C., Suckling D.M. (2013). Attractiveness and competitiveness of irradiated light brown apple moths. Entomol. Exp. Appl..

[12] Ikegawa Y., Ito K., Himuro C., Honma A. (2021). Sterile males and females can synergistically suppress wild pests targeted by sterile insect technique. J. Theor. Biol..

[13] Torres-Vila L.M., Facundo J.G., Rodríguez-Molina M.C., Stockel J. (2002). Heritable variation for female remating in *Lobesia botrana*, a usually monandrous moth. Anim. Behav..

[14] Lucchi A., Sambado P., Royo A.B.J., Bagnoli B., Benelli G. (2018). *Lobesia botrana* males mainly fly at dusk: Video camera-assisted pheromone traps and implications for mating disruption. J. Pest Sci..

[15] Guerfali M.M., Chevrier C. (2020). Determinant factors for sperm transfer and sperm storage within *Ceratitis capitata* (Diptera: Tephritidae) and impact on Sterile Insect Technique. J. Radiat. Res. Appl. Sci..

[16] Thiéry D., Moreau J. (2005). Relative performance of European grapevine moth (*Lobesia*
*botrana*) on grapes and other hosts. Oecologia.

[17] Ueno H., Ito Y. (1992). Sperm precedence in *Eysarcoris lewisi* Distant (Heteroptera: Pentatomidae) in relation to duration between oviposition and the last copulation. Appl. Entomol. Zool..

[18] Abacus Concepts (1994). StatView, Version 4.02.

[19] Makee H., Saour G. (2001). Factors influencing mating success, mating frequency, and fecundity in *Phthorimaea operculella* (Lepidoptera: Gelechiidae). Environ. Entomol..

[20] Makee H., Idris I., Hussian K. (2012). Factors influencing mating incidence and reproduction in codling moth *Cydia pomonella* L. (Lepidoptera: Tortricidae). Adv. Hort. Sci..

[21] Suckling D.M., Hackett J.K., Chhagan A., Barrington A., El-Sayed A.M. (2006). Effect of irradiation on female painted apple moth *Teia anartoides* (Lep., Lymantriidae) sterility and attractiveness to males. J. Appl. Entomol..

[22] Stepien T.L., Zmurchok C., Hengenius J.B., Rivera R.M.C., D’Orsogna M.R., Lindsay A.E. (2020). Moth mating: Modeling female pheromone calling and male navigational strategies to optimize reproductive success. Appl. Sci..

[23] Torres-Vila L.M., Rodríguez-Molina M.C., Stockel J. (2002). Delayed mating reduces reproductive output of female European grapevine moth, *Lobesia botrana* (Lepidoptera: Tortricidae). Bull Entomol. Res..

[24] Lalanne-Cassou B., Percy J., MacDonald J.A. (1977). Ultrastructure of sex pheromone gland cells in *Lobesia botrana* Den & Schiff. (Lepidoptera: Olethreutidae). Can. J. Zool..

[25] Tasin M., Bäckman A.-C., Bengtsson M., Varela N., Ioriatti C., Witzgall P. (2006). Wind tunnel attraction of grapevine moth females, *Lobesia botrana*, to natural and artificial grape odour. Chemoecology.

[26] Wang X.P., Fang Y.L., Zhang Z.N. (2011). Effects of delayed mating on the fecundity and longevity of females of diamondback moth, *Plutella xylostella*. Insect Sci..

[27] Makee H., Saour G. (2003). Noninherited sterility in irradiated *Phthorimaea operculella* females. J. Appl. Entomol..

[28] Zheng X.-L., Junyan L., Wen L., He X.Z., Wang Q. (2020). Mating delay reduces reproductive performance but not longevity in a monandrous moth. J. Insect Sci..

[29] Walker P.W., Allen G.R. (2010). Mating frequency and reproductive success in an income breeding moth, *Mnesampela private*. Entomol. Exp. Et App..

[30] Li Y.-Y., Yu J.-F., Lu Q., Xu J., Ye H. (2014). Female and male moths display different reproductive behavior when facing new versus previous mates. PLoS ONE.

[31] Xu J., Wang Q. (2010). Mechanisms of last male precedence in a moth: Sperm displacement at ejaculation and storage sites. Behav. Ecol..

[32] Milonas P.G., Partsinevelos G.K., Andow D.A. (2017). Effect of male mating history and age on remating by female European corn borer. PLoS ONE.

[33] Thorburn D.-M.J., Knell R.J., Parrett J.M. (2018). Sperm morph and remating frequency in the Indian meal moth, *Plodia interpunctella*. Biol. Lett..

[34] Xu J., Wang Q. (2014). Ejaculate economics: An experimental test in a moth. Biol. Lett..

[35] Seth R.K., Khan Z., Rao D.K., Zarin M. (2016). Flight activity and mating behavior of irradiated *Spodoptera litura* (Lepidoptera: Noctuidae) males and their F_1_ progeny for use of inherited sterility in pest management approaches. Fla. Entomol..

[36] Saour G., Makee H. (1999). Effect of gamma irradiation on sperm utilization in twice- mated female *Phthorimaea operculella* Zeller (Lep., Gelechiidae). J. Appl. Entomol..

[37] Suckling D.M., Conlong D.E., Carpenter J.E., Bloem K.A., Rendon P., Vreysen M.J.B. (2017). Global range expansion of pest Lepidoptera requires socially acceptable solutions. Biol. Invasions.

[38] Chapman R.F., Simpson S.J., Douglas A.E. (2013). The Insects: Structure and Function.

[39] Li H.-L., Wang X.-Y., Zheng X.-L., Lu W. (2020). Research progress on oviposition-related genes in insects. J. Insect Sci..

[40] Wainwright C., Sascha J.Y., Wilson D., Elliott M., Jukes A., Rosemary C.R. (2020). Phenology of the diamondback moth (*Plutella xylostella*) in the UK and provision of decision support for Brassica growers. Insects.

[41] Idris I., Hussian K., Alali N., Ikhtiar A. (2019). Irreversible fertility of irradiated *Phthorimaea operculella* (Lepidoptera: Gelechiidae) female. J. Bio Innov..

[42] Vreysen M.J.B., Hendrichs J., Enkerlin W.R. (2006). The sterile insect technique as a component of sustainable area-wide integrated pest management of selected horticulture insect pests. J. Fruit Ornam. Plant Res..

